# Effects of the Mediterranean Diet during pregnancy on the onset of allergy in at risk children: A study protocol of a multi-center, randomized- controlled, parallel groups, prospective trial (the PREMEDI study)

**DOI:** 10.3389/fnut.2022.951223

**Published:** 2022-10-13

**Authors:** Serena Coppola, Lorella Paparo, Lorenzo Chiariotti, Danilo Ercolini, Rita Nocerino, Anna Fiorenza de Giovanni di Santa Severina, Laura Carucci, Francesca De Filippis, Annalisa Agangi, Marcello Napolitano, Annalisa Passariello, Francesco Messina, Roberto Berni Canani

**Affiliations:** ^1^Department of Translational Medical Science, University of Naples Federico II, Naples, Italy; ^2^CEINGE Advanced Biotechnologies, University of Naples Federico II, Naples, Italy; ^3^Department of Molecular Medicine and Medical Biotechnology, University of Naples Federico II, Naples, Italy; ^4^Task Force on Microbiome Studies, University of Naples Federico II, Naples, Italy; ^5^Department of Agricultural Sciences, University of Naples Federico II, Naples, Italy; ^6^Neonatal Intensive Care Unit, Betania Evangelical Hospital, Naples, Italy; ^7^Department of Pediatric Cardiology, Monaldi Hospital, Naples, Italy

**Keywords:** food allergy, eczema, oculorhinitis, diet, epigenetics, microbiome

## Abstract

**Introduction:**

Maternal diet during pregnancy has been linked to offspring allergy risk and it could represent a potential target for allergy prevention. The Mediterranean Diet (MD) is considered one of the healthiest dietary models. Randomized-controlled trials on the effect of MD in preventing pediatric allergic diseases are still needed.

**Methods and analysis:**

The Mediterranean Diet during Pregnancy study (PREMEDI) will be a 9-month multi-center, randomized-controlled, parallel groups, prospective trial. Healthy women (20–35 years) at their first trimester of pregnancy at risk for atopy baby, will be randomly allocated to Group 1 (standard obstetrical and gynecological follow-up and nutritional counseling to promote MD) or Group 2 (standard obstetrical and gynecological follow-up alone). 138 mother-child pair per group will be needed to detect a reduction in cumulative incidence of ≥1 allergic disease at 24 months of age. The primary study aim will be the evaluation of the occurrence of allergic disorders in the first 24 months of life. The secondary aims will be the evaluation of maternal weight gain, pregnancy/perinatal complications, growth indices and occurrence of other chronic disorders, mother-child pair adherence to MD and gut microbiome features, breastfeeding duration and breast milk composition, epigenetic modulation of genes involved in immune system, and metabolic pathways in the offspring.

**Ethics and dissemination:**

The study protocol has been approved by the Ethics Committee of the University of Naples Federico II (number 283/21) and it will be conducted in accordance with the Helsinki Declaration (Fortaleza revision, 2013), the Good Clinical Practice Standards (CPMP/ICH/135/95), the Italian Decree-Law 196/2003 regarding personal data and the European regulations on this subject. The study has been registered in the Clinical Trials Protocol Registration System.

**Clinical trial registration:**

[http://clinicaltrials.gov], identifier [NCT05119868].

## Introduction

### Background and rationale

Prevalence and severity of pediatric allergic diseases are increasing steadily in the last two decades under the pressure of environmental factors acting on genetically predisposed individuals (1). Innovative strategies aimed at limiting the pediatric allergy burden are advocated, to reduce the socio-economic costs for the families and the healthcare systems (1). The first 1,000 days of life, from the conception to 24 months, are crucial to achieve long-term health outcomes and represent a strategic period to intervene under prevention and public health perspective (2).

Nutritional exposures during this critical period of life can influence the future disease susceptibility (3). Maternal diet during pregnancy has been linked to offspring allergy risk and it could represent a potential target for allergy prevention (1). The Mediterranean Diet (MD) is considered one of the healthiest dietary models, which impacts beneficially the gut microbiome (GM), providing high amounts of fiber, antioxidants polyphenols and vitamins, and a balanced ratio of essential fatty acids (ω6:ω3) (4). Notably, the MD beneficial effects are due to the synergistic and interactive combinations of nutrients, and the modulation of gene expression through epigenetic changes is one important mechanism by which MD can lead to regulatory effects in immune system (5). Advances in understanding diet-GM-epigenetics-immune system axis have highlighted the importance of GM-derived metabolites in maintaining health and immune homeostasis. Among the GM-derived metabolites, the short-chain fatty acids (SCFAs) have emerged as key signaling molecules driving beneficial downstream effects on immune pathways (6). MD is typified by a high-level of whole cereals, fruit, vegetables, and legumes, the main fermentation substrates for SCFAs microbial production (4). In addition, overall MD-derived components, like ω-3 long chain poly-unsaturated fatty acids such as eicosapentaenoic and docosahexaenoic acids derived from marine foodstuffs, mono-unsaturated oleic acid of olive oil (rich in antioxidants e.g., tocopherols), vitamins and trace elements, polyphenols and carotenoids including lycopene, are known for their anti-inflammatory and anti-allergic capacities, indicating their potencies to improve allergy protection by combining these elements (7).

Although, several observational birth-cohort and retrospective studies have provided conflicting results on the effects of MD in pregnancy on allergy prevention in the offspring. Eight studies have explored the effect of maternal MD during pregnancy on the incidence of allergic diseases in the offspring (8–15). Six of them were cohort studies (8–13), and two were cross sectional studies (14, 15). In the ALSPAC (Avon Longitudinal Study of Parents and Children) birth cohort study adherence to MD during pregnancy was not associated with a reduced risk of asthma or other allergic outcomes in the offspring but may be associated with increased small airway function in childhood (13). Similarly, a study involving 1,087 Spanish infants from the International Study of Wheezing in Infants (Estudio Internacional de Sibilancias en Lactantes, EISL) showed that the consumption of MD during pregnancy did not elicit protective effect for wheezing, recurrent wheezing, or eczema at 12 months of age in children (11). Another Spanish observational study did not evidence any association between maternal adherence to the MD and development of wheezing, rhinitis, and dermatitis in the children at 4 years of age (15). Likewise, the results of a USA pre-birth cohort of 1,376 mother-infant pairs from Project Viva showed that MD during pregnancy was not associated with recurrent wheeze at 3 years of age (12). Furthermore, a high adherence to MD during pregnancy was not significantly associated with reduction of infantile wheeze or eczema in two mother-child cohorts in Spain and Greece (10). On the contrary, two Spanish studies reported that high adherence to MD during pregnancy elicited significant negative associations with wheeze and atopy in children (8, 9), and one study from Mexico reported that adherence to MD was inversely associated with asthma, wheezing, rhinitis, sneezing, current sneezing, and current itchy-watery eyes (14). Lastly, a general low MD-adherence has been described during pregnancy (16). These data were confirmed by the results of a pilot study performed at our Center, where we observed a low adherence rate to MD in pregnant women at their first trimester of pregnancy (unpublished data).

Unfortunately, these studies have assessed maternal diet retrospectively. Thus, the lack of prospective randomized-controlled trials, exploring the effects of a specific nutritional counseling for MD promotion during pregnancy on allergy prevention in the offspring, is a major limitation for understanding the role of MD in pregnancy in preventing pediatric allergies. The Mediterranean Diet during Pregnancy study (PREMEDI) was designed to address this limitation.

Whilst previous studies provided conflicting results on the protective effect elicited by MD against pediatric allergies, it is unknown whether an intervention to promote the MD in pregnancy could reduce the likelihood of allergic diseases in the offspring. We hypothesize that a dietary intervention aiming to promote an optimal adherence to MD during pregnancy could be able to limit the occurrence of allergic diseases in early childhood, including atopic eczema, allergic oculorhinitis, allergic urticaria, asthma, and IgE and non-IgE mediated food allergies.

## Methods and analysis

### Study settings

The study will involve four tertiary gynecology and neonatology centers located in the Campania region in Italy. The annual number of births at each center is >1,500.

### Study design

The PREMEDI Study will be a 9-month multi-center, randomized-controlled, parallel groups, prospective trial.

The study will involve three parallel teams: Research Team (RT), Clinical Team (CT), and Statistical Team (ST). The RT will be composed by physicians, molecular biologists, and dietitians. The CT will be composed by gynecologists, neonatologists, pediatricians, family pediatricians, allergologists, dietitians, and pediatric nurses. The ST will be composed by statisticians. A Study Monitor will overview the trial’s data management and analysis.

The RT will be dedicated to the enrollment and the randomization of participants. Then, the dietitians of the RT will perform the personalized nutritional counseling during the study phases. The CT will evaluate the participants and will collect data in anonymous form and will deliver data to the ST. The ST, unaware of study cohorts, will review the study dataset and will undergo data cleaning and verification according to standard procedures. Finally, ST will lock the database once it will be declared complete and accurate and will perform statistical analysis.

**FIGURE 1:**
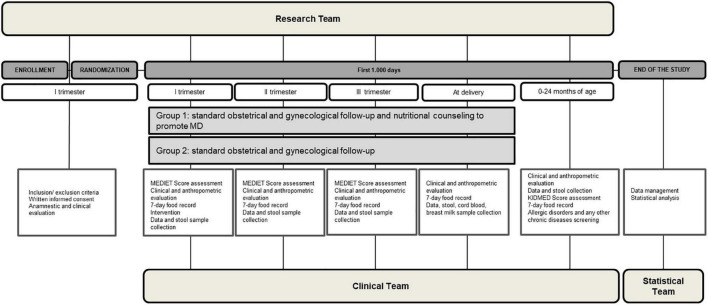
The study design. The figure shows a graphical representation of the study design, including the set of procedures at each study visit, and including the execution team.

The graphical representation of study design is reported in Figure 1.

**FIGURE 2:**
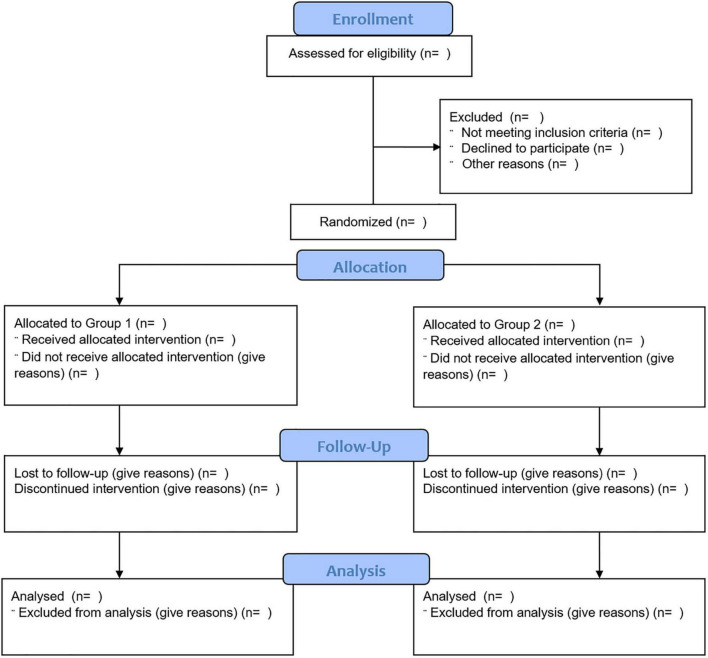
The study participants flow. The figure shows the flow of the participants through the study.

The Figure 2 reports the participant flow through the study.

**FIGURE 3:**
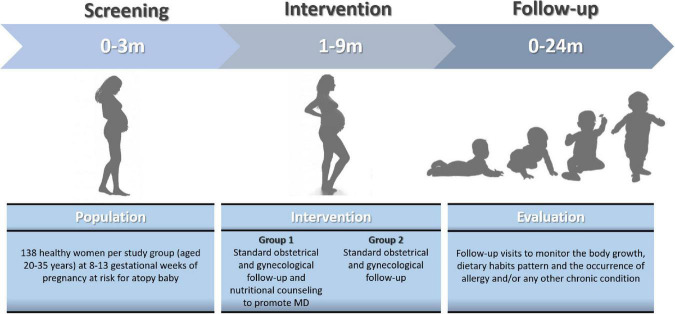
The study plan. The figure show the time schedule of screening, intervention, and follow-up.

In the Figure 3 is depicted the study plan.

### Eligibility criteria

We will consider for the study all healthy women (aged 20–35 years) attending their first pregnancy visit at 8–13 gestational weeks (GW) consecutively observed at the four centers. We will consider only pregnant women with a pregnancy of at risk for atopy baby. The atopy risk will be defined by the presence of at least one first degree family member of the baby with documented history of allergic diseases. We will exclude from the study all women aged <20 and >35 years, or with concomitant presence of infections, malignancies, major gastrointestinal malformations, immunodeficiencies, autoimmune diseases, chronic inflammatory bowel diseases, pre-existing diabetes, chronic renal disease, celiac disease, history of abdominal surgery with gastrointestinal resection, neurologic and neuropsychiatric disorders, genetic and metabolic disorders, vegan diet, twin pregnancy.

### Allocation and blinding

The randomization and sequence allocation will be performed according to a computer-generated randomization list by building a stratified randomization with permutated block-randomization, stratified by maternal body mass index (BMI) (<25, 25–29.9, and ≥30 kg/m^2^), and parity (1 or >1), in an allocation ratio of 1:1 in blocks of 4–6. The Group #1 will receive standard obstetrical and gynecological follow-up and nutritional counseling to promote MD provided by certified dietitians, and the Group #2: will receive standard obstetrical and gynecological follow-up alone. Due to the nature of the RCT design, participants and RT will be aware of the group assignment. Whereas the allocation to Group #1 or Group #2 will remain unknown to the CT and ST.

### Study outcomes

The main study outcome will be the evaluation of the effect of MD during pregnancy on the occurrence of allergic disorders at 24 months of age in the offspring.

Secondary study outcomes will include the evaluation of:

– women MD-adherence score (Med Diet Score);
– maternal weight gain and pregnancy complications (including gestational diabetes, arterial hypertension, gestosis, cholestasis, premature rupture of membranes (PROM), placental abruption, post-partum anemia, post-partum hemorrhage);
– perinatal complications (including intrauterine growth retardation (IUGR), small-for-gestational-age infant (SGA), large-for-gestational-age infant (LGA), preterm delivery, and fetal malformation) and neonatal birth weight;
– body growth indices and occurrence of infectious diseases and chronic non-transmissible conditions (obesity, autoimmune diseases, malignancies, neuropsychiatric disorders) in the first 2 years of age in the offspring;
– mother-child pair GM metagenomic and metabolomic features;
– exclusive and overall breastfeeding duration and breast milk composition;
– epigenetic modulation of genes involved in the regulation of immune system and metabolic pathways in the offspring;
– child MD-quality Index score (KIDMED).

### Interventions

All enrolled women will receive, by the CT gynecologists, recommendations about the importance of an adequate energy intake, optimal weight gain during pregnancy based on pre-pregnancy Body Mass Index (BMI) (17) and hygiene rules for the prevention of major foodborne illnesses during pregnancy according to the guidance provided by the Italian Ministry of Health (18). They were also recommended to walk ≥30 min/day. All enrolled women will be instructed on how to complete the 7-day food diary by the RT dietitians.

Only for women enrolled in the Group #1, a nutritional counseling will be provided by the RT dietitians. The RT dietitians will deliver the nutritional counseling over three face-to-face sessions, which will include a session at enrollment (8–13 GW), and two further sessions after 3 months from enrollment (14–28 GW) and after 6 months from enrollment (29–40 GW). The nutritional counseling will be based on a Mediterranean-style Diet. The key components of this diet will include the use of extra virgin olive oil as main cooking fat (at least four tablespoons/day), high intake of vegetables (two servings/day), fruits (three servings/day) (avoiding juices) and wholegrain cereals (three servings/day), three servings of skimmed dairy products/day, 2–3 servings of legumes/week, three servings of fish/week, three servings of nuts and seeds/week, drink at least two liters of water daily, a low consumption of red meat and processed meat, avoidance of refined grains, processed baked goods, pre-sliced bread, soft drinks and fresh juices, fast foods, and precooked meals.

Furthermore, after delivery, the CT will encourage women of both groups to breastfed wherever possible, to not avoid dietary food allergens during breastfeeding, to avoid the use of regular cow’s milk formula as supplementary feed for breastfed infants in the first week of life, and to introduce complementary feeding from 4 to 6 months of age, according to the European Academy of Allergy and Clinical Immunology guidelines for allergy prevention (19).

The decision to interrupt the study participation may take place at any time even without providing any explanation or justification, it will not entail any penalty, loss of benefits or loss of legal rights for mother-child pair and the mother-child personal data will always remain confidential.

### Data and sample collection

At baseline, after the first evaluation by the RT and the collection of written informed consent form, the CT will collect the following variables in a dedicated chart: anamnestic and clinical features, personal and anthropometric data, socio-demographic factors, gestational age, allergies, number of cohabitants, pets, sports activities, use of drugs, smoking exposure, education level, family and living conditions. At enrollment a validated questionnaire on MD-adherence (Med Diet Score) and a 7-day food diary (five weekdays and two weekend days) will be collected. The 7-day food dairy data will be analyzed using specific software (Winfood–Medimatica Srl) by a dietitian. The intake of drugs, dietary supplements, pre-, pro-, and synbiotics will be recorded in the same chart. Then, the RT will schedule for each participant follow-up visits every 12 weeks from the enrollment (8–13 GW), then at 14–28 GW and at 29–40 GW. During these visits the CT will perform a full physical examination, anamnestic and clinical data collection, stool sampling (at least 3 g), 7-day food diary collection, Med Diet Score assessment. Each item assigns a score of 0 or 1, with a total score ranging from 0 to 14. The three categories of adherence to MD are divided in ≤5 (low), 6–9 (medium), ≥10 (high) score. Furthermore, to ensure and encourage the completion of the 7-day food diary, one week before each visit, the RT will remind the women by a phone call to fill in the food diary daily for the following week and to return them at the following visit. At delivery a cord blood sample (at least 10 ml) and a breast milk sample (collected within 24 h after delivery) (3 ml) will be collected. All neonatal clinical features will be evaluated by CT. After delivery, for all babies a follow-up visit will be planned every 3 months for the first 12 months of life and then every 6 months until the age of 2 years. At each visit the following procedures will be adopted by CT: full anamnestic and clinical evaluation, body growth assessment, occurrence of allergic disorders, occurrence of other conditions, 7-day food diary, child Mediterranean Diet Quality Index score (KIDMED) evaluation, stool sampling (at least 3 g). At each visit the persistence of breastfeeding will be also assessed and a breast milk sample will be eventually collected (3 ml). In addition, Skin Prick Testing using main pediatric allergens will be performed at 12 and 24 months. All collected data and documents will always remain confidential.

### Evaluation of the occurrence of allergic disorders in the offspring

The occurrence of any allergic disorders in the offspring will be carefully monitored every 6 months for the first 2 years of life by the CT. During these visits, the CT will assess clinical status, body growth, occurrence of allergic symptoms. Unscheduled visits will be performed, if necessary, because of allergic symptoms or other morbidities. Whenever allergic symptoms or other morbidities occurred, parents will be instructed to contact the family pediatrician of the CT to have a medical examination of their child. At each visit, the CT will perform a full physical examination, and then, using standardized criteria, will decide on the atopic manifestation diagnosis (20, 21). The occurrence of atopic manifestation will be investigated, evaluating potential condition in differential diagnosis and the results of allergy screening tests. In case of discordance about an atopic manifestation diagnosis, further evaluation by another pediatric allergist, unaware of the study aims, will be performed. Atopic eczema will be diagnosed by pruritus, typical morphology, and distribution, a chronic or chronically relapsing course, and personal or family atopic history (three of four criteria), in addition to three minor criteria among a list of 21 as reported elsewhere (20). Allergic oculorhinitis will be diagnosed based on the symptoms of rhinitis, such as nasal congestion, sneezing, itching, rhinorrhea, current use of medication for these symptoms and/or conjunctivitis, after exclusion of infection. Allergic urticaria will be diagnosed if at least two episodes of itching eruptions or swelling with typical appearance will be observed by the parents or a physician and will be caused by the same allergen. The diagnosis of asthma in young children with a history of wheezing is more likely if they have: wheezing or coughing that occur with exercise, laughing or crying, or in the absence of an apparent respiratory infection; a history of other allergic disease (eczema or allergic rhinitis), allergen sensitization or asthma in first-degree relatives; clinical improvement during 2–3 months of controller treatment, and worsening after cessation (21). Alternative causes of recurrent wheezing will be considered and excluded. The occurrence of both IgE and non-IgE mediated food allergies will be evaluated using criteria previously adopted by others (22): convincing food allergy, based on the occurrence of suggestive clinical features of food allergy (urticaria, angioedema, atopic dermatitis, asthma, oculorhinitis, diarrhea, regurgitation/vomiting, abdominal pain, constipation, bloody stools, failure to thrive) after the ingestion of a particular food and clear response to the elimination diet; or confirmed food allergy, based on the presence of suggestive clinical features of food allergy, clear response to the elimination diet, positive food allergy screening tests (skin prick tests, serum specific IgE, atopy patch tests), and/or positive oral food challenge (OFC).

### Evaluation of other morbidities during the follow-up period

All babies will be carefully monitored during the study period (through scheduled or unscheduled visits that will be performed by the CT) for the occurrence of infectious diseases and/or chronic non-transmissible conditions (overweight/obesity, autoimmune diseases, malignancies, neuropsychiatric disorders). All diagnoses will be performed according to validated criteria. All data will be collected in a dedicated chart.

### Metagenomic and metabolomic analysis

All fecal and human milk samples will be stored at −80°C and subjected to microbial DNA extraction by RT according to the International Human Microbiome Standard Consortium (IHMSC) Standard Operating Procedures (23).

### Shotgun metagenomics analysis

DNA libraries will be sequenced on Illumina NovaSeq platform, leading to 2 × 150 bp, paired-end reads. Reads filtering will be performed using the Human Sequence Removal pipeline developed within the Human Microbiome Project by using the Best Match Tagger (24). Then, non-human reads will be quality-filtered using PRINSEQ (23): reads with bases having a Phred score <15 will be trimmed and those <75 bp will be discarded. High quality reads will be imported in MetaPhlAn3 (25) to obtain species-level, quantitative taxonomic profiles. Functional profiling will be obtained using HUMAnN3 (26). Genomes of dominant strains will be also reconstructed with a pipeline recently validated (27) and compared, to define if a strain-level variation exists upon intervention.

### Metabolomic analysis

One gram of frozen feces and 1 ml of breast milk will be diluted with saline buffer, vortexed and centrifuged (12,000 × *g*) for 10 min in 2 ml tubes for SCFAs determination. The supernatants will be filtered (0.45 μm) and stored at −20°C until analysis. The extracts will be acidified with 20 μl of 85% (w/v) phosphoric acid and 0.5 ml of ethyl acetate, mixed, centrifuged (12,000 × *g*) for 1 h, and extracted in duplicate. About 0.5 ml of the pooled extract containing the acidified SCFAs will be transferred into a 2 ml glass vial and loaded onto an Agilent Technologies (Santa Clara, CA, USA) 7,890 gas chromatograph (GC) system with automatic loader/injector, combined with Mass Spectrometry (MS). The GC-MS column will be an Agilent 122-7,032 ui (DB-WAX-U, Agilent Technologies, Santa Clara, CA, USA) of 30 m, internal diameter of 0.25 mm, and film thickness of 0.25 μm. The GC-MS will be programmed to achieve the following run parameters: initial temperature of 50°C, hold of 1 min, ramp of 10°C min^–1^ up to a final temperature of 180°C, total run time of 20 min, gas flow of 70 ml min^–1^ splitless to maintain 12.67 p.s.i. column head pressure, and septum purge of 2.0 ml min^–1^. Helium will be the carrier gas (1.5 ml min^–1^ constant). Parameters of mass spectrometer will be source at 230°C and MS Quad at 150°C. The GraphPad PRISM eight program will be used to determine the concentration in mm. The data will be inserted in the “XY” form in which in the “X” frame the values of the straight concentration-response were reported, while in the “Y” box the values of the area under the curve (AUC) related to the peaks obtained from the mass gas will be reported. The AUC values of the single samples (obtained from the mass gas) will be interpolated with the line X (concentration-response) to determine the corresponding mm.

### Epigenetic analyses

Emerging data support the concept that epigenetic mechanisms could drive the occurrence of allergic diseases. Epigenetic biomarkers have been pointed out, suggesting the role of selected methylation rate of the promoter region of Th1 and Th2 cytokine genes and of selected microRNAs expression. We will investigate these biomarkers with targeted DNA methylation analysis with the aim to link the MD adherence with the expression of these biomarkers and clinical outcome also through mediation analysis. Parallelly, we will attempt at epigenome-wide evaluation to explore the potential role of other epigenetic mechanism elicited by MD during pregnancy as protective action against allergy.

Cord blood will be collected by CT team in two EDTA-tubes (5 ml/each) and immediately transported to the laboratory for DNA and RNA extraction using the Extraction Kit (GE Healthcare, Chicago, IL, USA) performed by RT. One microgram of extracted DNA will be modified with sodium bisulfite to convert all unmethylated, but not methylated-cytosine to uracil. Bisulfite conversion will be carried out using the EZ DNA Methylation Gold Kit (ZYMO Research Co., Orange, CA, USA), according to the manufacturer’s instructions. The converted DNA will be stored at −80°C until used.

Targeted DNA methylation analysis of Th1 and Th2 cytokine genes will be performed by next-generation sequencing. Briefly, after the bisulfite treatment, a first step of PCR will be performed using primes generating amplicons of 300–400 bp. These primers will contain overhang adapters sequences at each 5’end (FW: 5′-TCGTCGGCAGCGTCAGATGTGTATAAGAGACAG-3′; RV: 5′-GTCTCGTGGGCTCGGAGATGTGTATAAGAGACAG-3′) that will be recognized in the second step of PCR. The first PCR conditions will be: one cycle at 95°C for 2 min followed by 32 cycles at 95°C for 30 s, [primer Tm] for 40 s, 72°C for 50 s, followed by a final extension step at 72°C for 6 min. To eliminate the excess of primers a first purification step will be performed by using AMPure magnetic Beads (Beckman-Coulter, Brea, CA, USA) with a ratio AMPure Beads/PCR products of 0.8. Then, a second step of PCR will be performed to add multiplexing indices and Illumina sequencing adapters. The condition of second PCR step will be: one cycle at 95°C for 2 min followed by eight cycles at 95°C 30 s, 55°C for 40 s, 72°C for 40 s, followed by a final extension step at 72°C for 5 min. Another purification with AMPure Beads was performed with a ratio Beads/PCR of 1.2. The quantification process and the check of amplicon quality will be performed respectively using Qubit^®^ 2.0 Fluorometer and Agilent 2100 Bioanalyzer DNA 1,000 Kit (Agilent Technologies, Santa Clara, CA, USA), according to the manufacturer’s instructions. The amplicon bisulfite library will be prepared. Phix control libraries (Illumina) [15% (v/v)] will be used to increase the diversity of base calling during sequencing. The sequencing will be performed with V2 reagents kits on Illumina MiSeq system (Illumina, San Diego, CA, USA). Paired-end sequencing was carried out in 251 cycles per read (251 × 2). Paired-end reads obtained from Illumina Miseq platform will be first assembled using PEAR tool with a minimum of 40 overlapping residues as threshold. The obtained sequences were analyzed using ampliMethProfiler pipeline software^1^ specifically designed for deep-targeted amplicon bisulfite sequencing (28). AmpliMethProfiler produces quality filtered FASTA files for each sample and directly extracts methylation average values and methylation profiles.

Global methylome analysis will be performed using Epigenome-Wide Association Study (EWAS) approach. Infinium TM Methylation EPIC Bead Chip by Illumina (San Diego, CA, USA) will be used to explore over 8,50,000 methylation sites per sample at single-nucleotide resolution, according to the manufacturer’s protocol.

For miRNA expression analysis, RNA will be extracted from the second EDTA tubes using Trizol protocol (Invitrogen, Life Technologies Europe BV, Monza, Italy) and TaqMan^®^ Array Human MicroRNA A Card v2.0, contains 384 TaqMan^®^ MicroRNA Assays, enabling accurate quantization of 377 human microRNAs (Applied Biosystem) will be used.

### Data entry

The CT will enter all collected data in a case report form using a single data-entry method. All data recorded in the case report form will be entered in the study database anonymously by the same researcher of CT staff. Study monitoring will be performed by an independent clinical trial monitor and will include on-site visits and telephone communications. The monitor will review the clinical forms for completeness, clarity and consistency and will instruct the RT personnel to make any corrections or additions. Then, the ST unaware of study cohorts will review the study dataset and will undergo data cleaning and verification according to standard procedures. Finally, ST will lock the database once it will be declared complete and accurate and the statistical analysis will be performed.

### Sample size calculation

Based on the results of the EUROPREVALL cohort (29), reporting a cumulative incidence for allergic diseases of 30%, and on the results of a previous pilot study performed at our center (unpublished data) showing a drop-out rate to this kind of intervention up to 15%, we estimated that 138 mother-child pair per group will be needed to detect a reduction in cumulative incidence of at least one allergic disease at 24 months from 30 to 15%, with a power of 0.80 at an alpha level of 0.05 (Pearson’s Chi-square, two-tailed test).

### Statistical methods

All data will be collected in a dedicated database and analyzed by the ST. We will perform intention to treat (ITT) and per-protocol analysis. We will perform ITT analysis by considering the mother-child pair lost after randomization: (1) all missing values of the primary outcome will set to the worst outcome in both groups (equal-case scenario ITT) and (2) missing values of the primary outcome will set to the worst outcome in the Group 1 and to the best outcome in the Group 2 (worst-case scenario ITT). The worst outcome will be defined as the occurrence of ≥1 atopic condition at 24 months, in the offspring; the best outcome will be its opposite. The secondary outcomes will be evaluated using per-protocol analysis. The Kolmogorov-Smirnov test will be used to determine whether variables are normally distributed. Descriptive statistics will be used to summarize baseline characteristics. The Student *t*-test will be used to compare mean values of continuous variables for approximating a normal distribution. For non-normally distributed variables, the Mann-Whitney *U* test will be used. The χ^2^ test or Fisher exact test will be used, when appropriate. We will use a binomial regression model (BRM) to estimate the incidence of the main outcome, i.e., at least one atopic condition at 24 months, in the offspring. The response variable of the BRM will be the presence of at least one atopic condition at 24 months (0 = no; 1 = yes), and the predictor will be the treatment cohort (0 = Group 1; 1 = Group 2). To evaluate the effect of potential confounders on the main outcome, we will add each of them separately to the aforementioned BRM and we will evaluate the changes in the estimated risk ratios. The evaluated potential confounders will be sex (0 = female; 1 = male), age (months), cesarean delivery (0 = no; 1 = yes), born at term (0 = no; 1 = yes), breastfed for at least 2 months (0 = no; 1 = yes), weaning (months), siblings (number), exposed to passive smoking (0 = no; 1 = yes), mother smoked during pregnancy (0 = no; 1 = yes), and exposed to pets (0 = no; 1 = yes), drug use (0 = no; 1 = yes), antiseptics exposure (0 = no; 1 = yes), lifestyle (0 = rural; 1 = urban), dietary habits (0 = low MD-adherence; 1 = medium MD-adherence; 2 = high MD-adherence) and pollution exposure (0 = no; 1 = yes). BRM is among the most commonly suggested methods to report the results from randomized controlled trials with binary outcomes. Regression modeling allows adding baseline co-variables to increase the precision of the estimate and better control for potential confounders. Comparisons of taxa or gene abundance between groups will be carried out using pairwise Wilcoxon tests. Statistical significance of pangene prevalence will be verified through Fisher’s test with multiple-hypothesis testing corrections *via* the false discovery rate (FDR). Machine learning-based classification analysis will be done using the MetAML package (30) and by considering random forests (RFs) as back-end classifier for all the results. Results will be obtained through a fivefold cross-validation and averaged on 20 independent runs. To evaluate whether the significant associations for the cord blood analysis primarily reflect maternal adherence to MD, additional models adjusted for cord blood DNA methylation of the same CpG site and for miRNAs will be evaluated when examining the association between DNA methylation/miRNAs and childhood allergic sensitization. For DNA methylation, using a reference methylation Quantitative Trait Loci (QTL) library, we will evaluate the genetic control of methylation for the top 30 associated CpG sites identified from the mid-childhood meta-analysis for atopic sensitization, environmental and food allergen sensitization. We leveraged genetic-epigenetic information from the ARIES large-scale genome-mQTL analysis, which included 1,000 mother-child pairs^2^ (31). mQTL comparisons will be focused on the *cis* position (i.e., a genetic variant within ±500 Kb of a methylation locus) measured from cord blood, and with MAF ≥5%. The level of significance for all statistical tests will be two-sided, *p* < 0.05. An integrative approach that combines multi-omics data together with integrated clinical data analysis will be performed. Multivariable linear regression will be used to analyze associations differentially methylated regions in cord blood DNA with clinical measures of offspring. All significance values will be corrected to take into account the effect of multiple comparisons, using the Bonferroni adjustment. All analyses will be performed with Stata 16.1 (Stata Corporation, College Station, TX, USA), SAS^®^ version 9.4 (SAS Institute, Inc., Cary, NC, USA) and R version 3.4.1.

### Retention plan

The four tertiary centers involved in the PREMEDI study will host gynecology, neonatology, and pediatrics facilities to perform on site the follow-up visits. Furthermore, the collaboration with family pediatricians (who in the Italian Health Care System provide primary care of patients from birth to 16 years), will facilitate an optimal adherence to all study phases.

## Discussion

Allergic diseases are major chronic non-transmissible conditions for the Italian pediatric population.

Mediterranean Diet, during pregnancy and first 2 years of life, has been proposed as potential strategy to prevent several conditions in the pediatric age, including allergic diseases (32). Unfortunately, the lack of prospective randomized-controlled trials, exploring the effects of a specific nutritional counseling for MD promotion during pregnancy on allergy prevention in the offspring, is a major limitation for understanding the efficacy of MD in pregnancy in preventing pediatric allergies. The PREMEDI study was designed to address this limitation.

Strengths and limitations of this study:

– The PREMEDI study is the first multi-center, randomized-controlled, prospective trial to evaluate the effect of a specific nutritional counseling for MD promotion in pregnancy on the onset of allergies in the offspring;
– The PREMEDI study is inspired by solid preclinical and clinical evidence and involves a multidisciplinary team (composed by gynecologists, neonatologists, pediatricians, allergists, dietitians, pediatric nurses, microbiologists, biologists, biostatisticians) experienced in pediatric allergy;

– The reduction in cumulative incidence of allergic diseases in the pediatric population could have a positive impact on the socio-economic costs for the families and Healthcare System for the management of these conditions;
– The PREMEDI trial has limited risks, given the lack of invasive procedures, and the expertise of RT;
– One possible pitfall of the study could derive from the fact that the trial will be conducted in a population with similar ethnicity and genetic background, demographic features, and environmental factors exposure. Thus, the results of the trial should be verified in populations with different features;
– Another possible limitation of the study may be the lack of evaluation of objective biomarkers of MD-adherence, such as plasma or urinary levels of selected nutrients.

## Ethics and dissemination

The first version of the study protocol, the information sheet, the informed consent form, and the clinical chart have been reviewed and approved on October 11th, 2021, by the Ethical Committee of the University of Naples Federico II (protocol identification number 283/21). The study will be conducted in accordance with the Helsinki Declaration (Fortaleza revision, 2013), the Good Clinical Practice Standards (CPMP/ICH/135/95), the Italian Decree-Law 196/2003 regarding personal data, and the European regulations on this subject. The study has been registered in the Clinical Trials Protocol Registration System http://clinicaltrials.gov with the number identification ID NCT05119868. Any study protocol modifications (e.g., changes to eligibility criteria, outcomes, analyses) will be promptly communicated to relevant parties (e.g., investigators, REC/IRBs, trial participants, trial registries, journals, regulators). The RT will track the manuscript in accordance with authorship guidelines. There are no plans for the use of professional writers. The results of this study will be disseminated through presentation at scientific conferences and publication in peer-reviewed journals. If women enrolled will express interest in learning the results of the study, the RT staff will provide an abstract of the study results on completion of data collection.

## Author contributions

SC, AFDGDSS, LaC, AA, MN, AP, FM, and RBC assessed the eligibility (inclusion/exclusion criteria), collected written informed consent, performed anamnestic and clinical evaluation, collected data in a case report form, entered anonymously the data into the study database, collected biological samples (stool, breast milk, cord blood), and performed the interventions. LP, LoC, DE, and FD conducted laboratory analyses on fecal, blood, and breast milk samples. RN wrote the statistical methods. All authors substantially contributed to the development of conception and design of the present trial, involved in drafting the manuscript of this study protocol or revising it critically for important intellectual content, and approved the final manuscript.

## Footnotes

1
https://sourceforge.net/projects/amplimethprofiler

2
http://www.mqtldb.org/

## References

[1] Venter C Agostoni C Arshad SH Ben-Abdallah M Du Toit G Fleischer DM Dietary factors during pregnancy and atopic outcomes in childhood: A systematic review from the European academy of allergy and clinical immunology. *Pediatr Allergy Immunol.* (2020) 31:889–912. 10.1111/pai.13303 32524677 PMC9588404

[2] Vandenplas Y. Early life and nutrition and allergy development. *Nutrients.* (2022) 14:282. 10.3390/nu14020282 35057463 PMC8779902

[3] Moreno Villares JM Collado MC Larqué E Leis Trabazo R Saenz De Pipaón M Moreno Aznar LA. The first 1000 days: An opportunity to reduce the burden of noncommunicable diseases. *Nutr Hosp.* (2019) 36:218–32. 10.20960/nh.02453 30836758

[4] De Filippis F Pellegrini N Vannini L Jeffery IB La Storia A Laghi L High-level adherence to a Mediterranean diet beneficially impacts the gut microbiota and associated metabolome. *Gut.* (2016) 65:1812–21. 10.1136/gutjnl-2015-309957 26416813

[5] Del Chierico F Vernocchi P Dallapiccola B Putignani L. Mediterranean diet and health: Food effects on gut microbiota and disease control. *Int J Mol Sci.* (2014) 15:11678–99. 10.3390/ijms150711678 24987952 PMC4139807

[6] Tan J McKenzie C Vuillermin PJ Goverse G Vinuesa CG Mebius RE Dietary fiber and bacterial SCFA enhance oral tolerance and protect against food allergy through diverse cellular pathways. *Cell Rep.* (2016) 15:2809–24. 10.1016/j.celrep.2016.05.047 27332875

[7] Hogenkamp A Ehlers A Garssen J Willemsen L. Allergy modulation by N-3 long chain polyunsaturated fatty acids and fat soluble nutrients of the mediterranean diet. *Front Pharmacol.* (2020) 11:1244. 10.3389/fphar.2020.01244 32973501 PMC7472571

[8] Chatzi L Torrent M Romieu I Garcia-Esteban R Ferrer C Vioque J Mediterranean diet in pregnancy is protective for wheeze and atopy in childhood. *Thorax.* (2008) 63:507–13. 10.1136/thx.2007.081745 18198206

[9] Castro-Rodriguez JA Garcia-Marcos L Sanchez-Solis M Pérez-Fernández V Martinez-Torres A Mallol J. Olive oil during pregnancy is associated with reduced wheezing during the first year of life of the offspring. *Pediatr Pulmonol.* (2010) 45:395–402. 10.1002/ppul.21205 20306538

[10] Chatzi L Garcia R Roumeliotaki T Basterrechea M Begiristain H Iñiguez C Mediterranean diet adherence during pregnancy and risk of wheeze and eczema in the first year of life: INMA (Spain) and RHEA (Greece) mother-child cohort studies. *Br J Nutr.* (2013) 110:2058–68. 10.1017/S0007114513001426 23680284

[11] Alvarez Zallo N Aguinaga-Ontoso I Alvarez-Alvarez I Marin-Fernandez B Guillén-Grima F Azcona-San Julián C. Influence of the Mediterranean diet during pregnancy in the development of wheezing and eczema in infants in Pamplona. *Spain. Allergol Immunopathol.* (2018) 46:9–14. 10.1016/j.aller.2017.02.009 28629669

[12] Lange NE Rifas-Shiman SL Camargo CA Jr. Gold DR Gillman MW Litonjua AA. Maternal dietary pattern during pregnancy is not associated with recurrent wheeze in children. *J Allergy Clin Immunol.* (2010) 126:250.e–5.e. 10.1016/j.jaci.2010.05.009 20584543 PMC2917539

[13] Bédard A Northstone K Henderson AJ Shaheen SO. Mediterranean diet during pregnancy and childhood respiratory and atopic outcomes: Birth cohort study. *Eur Respir J.* (2020) 55:1901215. 10.1183/13993003.01215-2019 31831586 PMC7066469

[14] de Batlle J Garcia-Aymerich J Barraza-Villarreal A Antó JM Romieu I. Mediterranean diet is associated with reduced asthma and rhinitis in Mexican children. *Allergy.* (2008) 63:1310–6. 10.1111/j.1398-9995.2008.01722.x 18782109

[15] Castro-Rodriguez JA Ramirez-Hernandez M Padilla O Pacheco-Gonzalez RM Pérez-Fernández V Garcia-Marcos L. Effect of foods and Mediterranean diet during pregnancy and first years of life on wheezing, rhinitis and dermatitis in preschoolers. *Allergol Immunopathol.* (2916) 44:400–9. 10.1016/j.aller.2015.12.002 27087566

[16] Orihara K Haraguchi A Shibata S. Crosstalk among circadian rhythm, obesity and allergy. *Int J Mol Sci.* (2020) 21:1884. 10.3390/ijms21051884 32164209 PMC7084300

[17] Institute of Medicine (Us) and National Research Council (Us) Committee to Reexamine Iom Pregnancy Weight Guidelines. In: Rasmussen KM Yaktine AL editors. *Weight gain during pregnancy: Reexamining the guidelines.* Washington, DC: National Academies Press (2009). 20669500

[18] Ministero della Salute. *La Quarta Dose Per Gli Over 60 E Fondamentale*. (2022). Available online at: https://www.salute.gov.it/ (accessed February 23, 2022).

[19] Halken S Muraro A de Silva D Khaleva E Angier E Arasi S EAACI guideline: Preventing the development of food allergy in infants and young children (2020 update). *Pediatr Allergy Immunol.* (2021) 32:843–58. 10.1111/pai.13496 33710678

[20] Nocerino R Bedogni G Carucci L Cosenza L Cozzolino T Paparo L The impact of formula choice for the management of pediatric cow’s milk allergy on the occurrence of other allergic manifestations: The atopic march cohort study. *J Pediatr.* (2021) 232:183.e–91.e. 10.1016/j.jpeds.2021.01.059 33524387

[21] Reddel HK Bacharier LB Bateman ED Brightling CE Brusselle GG Buhl R Global initiative for asthma strategy 2021: Executive summary and rationale for key changes. *Eur Respir J.* (2021) 59:2102730. 10.1183/13993003.02730-2021 34667060 PMC8719459

[22] Valerio G Maffeis C Saggese G Ambruzzi MA Balsamo A Bellone S Diagnosis, treatment and prevention of pediatric obesity: Consensus position statement of the Italian society for pediatric endocrinology and diabetology and the Italian society of pediatrics. *Ital J Pediatr.* (2018) 44:88. 10.1186/s13052-018-0525-6 30064525 PMC6069785

[23] The International Human Microbiome Standards. *Seventh Framework Programme*. (2022). Available online at: www.microbiome-standards.org (accessed February 23, 2022).

[24] Sherry S. *Human sequence removal national center for biotechnology information.* (2022). Available online at: https://hmpdacc.org/hmp/doc/HumanSequenceRemoval_SOP.pdf (accessed February 23, 2022).

[25] Schmieder R Edwards R. Quality control and preprocessing of metagenomic datasets. *Bioinformatics.* (2011) 27:863–4. 10.1093/bioinformatics/btr026 21278185 PMC3051327

[26] Beghini F McIver LJ Blanco-Míguez A Dubois L Asnicar F Maharjan S Integrating taxonomic, functional, and strain-level profiling of diverse microbial communities with bioBakery 3. *eLife.* (2021) 10:e65088. 10.7554/eLife.65088 33944776 PMC8096432

[27] Franzosa EA McIver LJ Rahnavard G Thompson LR Schirmer M Weingart G Species-level functional profiling of metagenomes and metatranscriptomes. *Nat Methods.* (2018) 15:962–8. 10.1038/s41592-018-0176-y 30377376 PMC6235447

[28] Scala G Affinito O Palumbo D Florio E Monticelli A Miele G ampliMethProfiler: A pipeline for the analysis of CpG methylation profiles of targeted deep bisulfite sequenced amplicons. *BMC Bioinformatics.* (2016) 17:484. 10.1186/s12859-016-1380-3 27884103 PMC5123276

[29] McBride D Keil T Grabenhenrich L Dubakiene R Drasutiene G Fiocchi A The EuroPrevall birth cohort study on food allergy: Baseline characteristics of 12,000 newborns and their families from nine European countries. *Pediatr Allergy Immunol.* (2012) 23:230–9. 10.1111/j.1399-3038.2011.01254.x 22192443

[30] Pasolli E Truong DT Malik F Waldron L Segata N. Machine learning meta-analysis of large metagenomic datasets: Tools and biological insights. *PLoS Comput Biol.* (2016) 12:e1004977. 10.1371/journal.pcbi.1004977 27400279 PMC4939962

[31] Gaunt TR Shihab HA Hemani G Min JL Woodward G Lyttleton O Systematic identification of genetic influences on methylation across the human life course. *Genome Biol.* (2016) 17:61. 10.1186/s13059-016-0926-z 27036880 PMC4818469

[32] Castro-Rodriguez JA Garcia-Marcos L. What are the effects of a mediterranean diet on allergies and asthma in children? *Front Pediatr.* (2017) 5:72. 10.3389/fped.2017.00072 28484688 PMC5399020

